# Unraveling the surface proteomic profile of multiple myeloma to reveal new immunotherapeutic targets and markers of drug resistance

**DOI:** 10.15698/cst2022.11.273

**Published:** 2022-10-13

**Authors:** Bonell Patiño-Escobar, Ian D. Ferguson, Arun P. Wiita

**Affiliations:** 1 Dept. of Laboratory Medicine, University of California, San Francisco, CA, USA; 2 Program in Cancer Biology, Stanford University School of Medicine, CA, USA; 3 Dept. of Bioengineering and Therapeutic Sciences, University of California, San Francisco, CA, USA; 4 Chan Zuckerberg Biohub, San Francisco, CA, USA

**Keywords:** myeloma, immunotherapy, proteomics, surfaceome, drug resistance, CAR-T

## Abstract

The cell surface proteome (“surfaceome”) serves as the interface between diseased cells and their local microenvironment. In cancer, this compartment is critical not only for defining tumor biology but also serves as a rich source of potential therapeutic targets and diagnostic markers. Recently, we profiled the surfaceome of the blood cancer multiple myeloma, an incurable plasma cell malignancy. While available small molecule agents can drive initial remissions in myeloma, resistance inevitably occurs. Several new classes of immunotherapies targeting myeloma surface antigens, including antibody therapeutics and chimeric antigen receptor (CAR) T-cells, can further prolong survival. However, new approaches are still needed for those who relapse. We thus applied the glycoprotein cell surface capture (CSC) methodology to panel of multiple myeloma cell lines, identifying key surface protein features of malignant plasma cells. We characterized the most abundant surface proteins on plasma cells, nominating CD48 as a high-density antigen favorable for a possible avidity-based strategy to enhance CAR-T efficacy. After chronic resistance to proteasome inhibitors, a first-line therapy, we found significant alterations in the surface profile of myeloma cells, including down-regulation of CD50, CD361/EVI2B, and CD53, while resistance to another first-line therapy, lenalidomide, drove increases in CD33 and CD45/PTPRC. In contrast, short-term treatment with lenalidomide led to upregulation of the surface antigen MUC-1, thereby enhancing efficacy of MUC-1 targeting CAR-T cells. Integrating our proteomics data with available transcriptome datasets, we developed a scoring system to rank potential standalone immunotherapy targets. Novel targets of interest included CCR10, TXNDC11, and LILRB4. We developed proof-of-principle CAR-T cells versus CCR10 using its natural ligand, CCL27, as an antigen recognition domain. Finally, we developed a “miniaturized” version of the CSC methodology and applied it to primary myeloma patient specimens. Overall, our work creates a unique resource for the myeloma community. This study also supports unbiased surface proteomic profiling as a fruitful strategy for identifying new therapeutic targets and markers of drug resistance, that could have utility in improving myeloma patient outcomes. Similar approaches could be readily applied to additional tumor types or even models/tissues derived from other diseases.

The armamentarium of myeloma therapies has dramatically expanded over the past two decades. Treatments now available include proteasome inhibitors, immunomodulatory agents (thalidomide analogs), surface antigen targeted immunotherapies (monoclonal antibodies, antibody-drug conjugates, bispecific antibodies, and CAR-T cells), and more. While median survival post-diagnosis has increased from less than 2 years to nearly 10 years during this time, myeloma still unfortunately remains incurable. Myeloma patients with relapsed/refractory disease, and particularly those relapsing after current immunotherapies, continue to present a critical unmet need.

To address this problem, several groups have analyzed transcriptome datasets to identify new myeloma immunotherapy targets. However, in general, transcriptome abundance may only weakly correlate with surface protein abundance. Thus, mRNA-only target discovery efforts provide only an incomplete picture of the “surfaceome”. In contrast, flow cytometry or mass cytometry/CYTOF are powerful techniques to directly measure protein abundance at the tumor cell surface. However, these methods are limited to a maximum of ∼40 previously known antigens and require available high-quality antibodies. Therefore, these protein-level approaches are not appropriate for discovery of novel targets.

To overcome these limitations, our group utilizes the strategy of relatively unbiased cell surface proteomics by mass spectrometry. Our modified glycoprotein cell surface capture (CSC) method first involves using cell-impermeant reagents to oxidize and biotinylate cell surface *N*-linked glycoproteins on live cell. This surface protein labeling is followed by enrichment on streptavidin beads and on-bead proteolysis to process samples to peptides as needed for mass spectrometry. In our work, we first applied this strategy to four different myeloma cell line models (KMS-12PE, AMO1, RPMI-8226, L363), using inputs of ∼30e6 cells per replicate. Across lines, we identified 1245 proteins annotated as membrane-bound in Uniprot, of which 530 are localized to the cell surface with high confidence. Validating our strategy, we identified nearly all known canonical diagnostic markers and immunotherapeutic targets in myeloma, including BCMA, CD138/SDC1, CD38, CD56, SLAMF7/CS-1, CD46, Integrin-b7 (ITGB7), CD74/HLA-DR, TACI, CD48/ SLAMF2, and LY9/CD229. These initial findings already expand the known universe of myeloma cell surface antigens.

To delve into this resource, we first analyzed our proteomic data to identify the most abundant proteins present on the myeloma plasma cell surface. Based on the intensity of mass spectrometric signal, we identified the most abundant proteins as 4F2 (encoded by *SLC3A2* gene) and LAT1 (*SLC7A5*), which comprise the heterodimeric large neutral amino acid transporter CD98. This finding emphasizes the centrality of protein metabolic requirements for plasma cell survival. Other high abundance proteins were related to maintaining cellular homeostasis or cellular signaling (**Fig. 1**). These data provide the first insight into the proteins that most predominate on the myeloma surface proteomic landscape and suggest key nodes of myeloma plasma cell biology.

**Figure 1:**
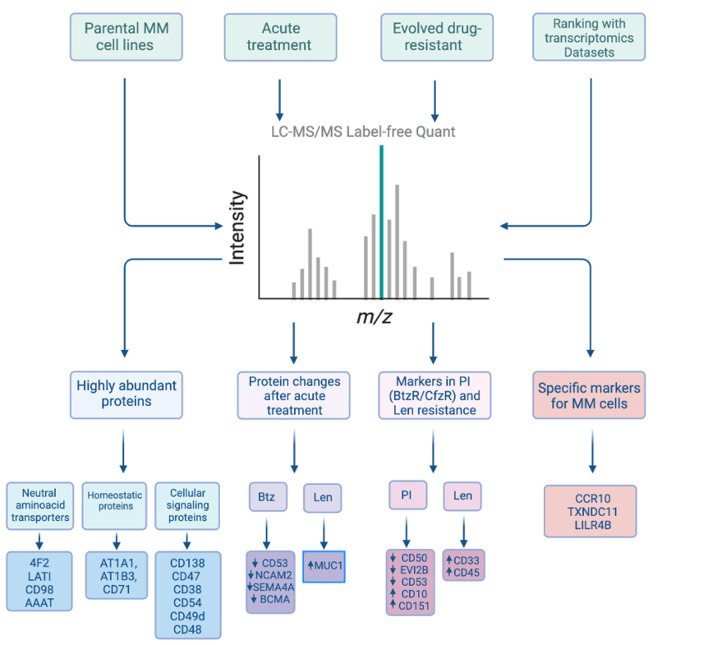
FIGURE 1: Overview of analyses performed to identify surface protein markers reflective of the baseline myeloma cell proteome as well as in response to therapeutic exposure. Figure created with BioRender.

Of these antigens, CD38 and CD48 are intriguing as they are known to be exclusively expressed on hematopoietic cells. We hypothesized that such highly abundant myeloma surface antigens may be favorable for an avidity-based strategy to enhance CAR-T cell therapy. Specifically, in this “locking on” approach, we could take advantage of the high abundance of these antigens to increase BCMA-targeting CAR-T cell dwell time on a tumor cell, thereby providing more opportunity for antigen-selective killing (**Fig. 2A**). Therefore, we used calibrated flow cytometry on both cell lines and primary myeloma bone marrow aspirate samples from relapsed/refractory myeloma patients to assess CD38 and CD48 density. While both markers are expressed at high copy number, consistent with proteomic findings, CD48 showed the higher density (range: 59,307–2,769,932 copies/cell), suggesting it as a promising target for this proposed “locking on” strategy.

**Figure 2:**
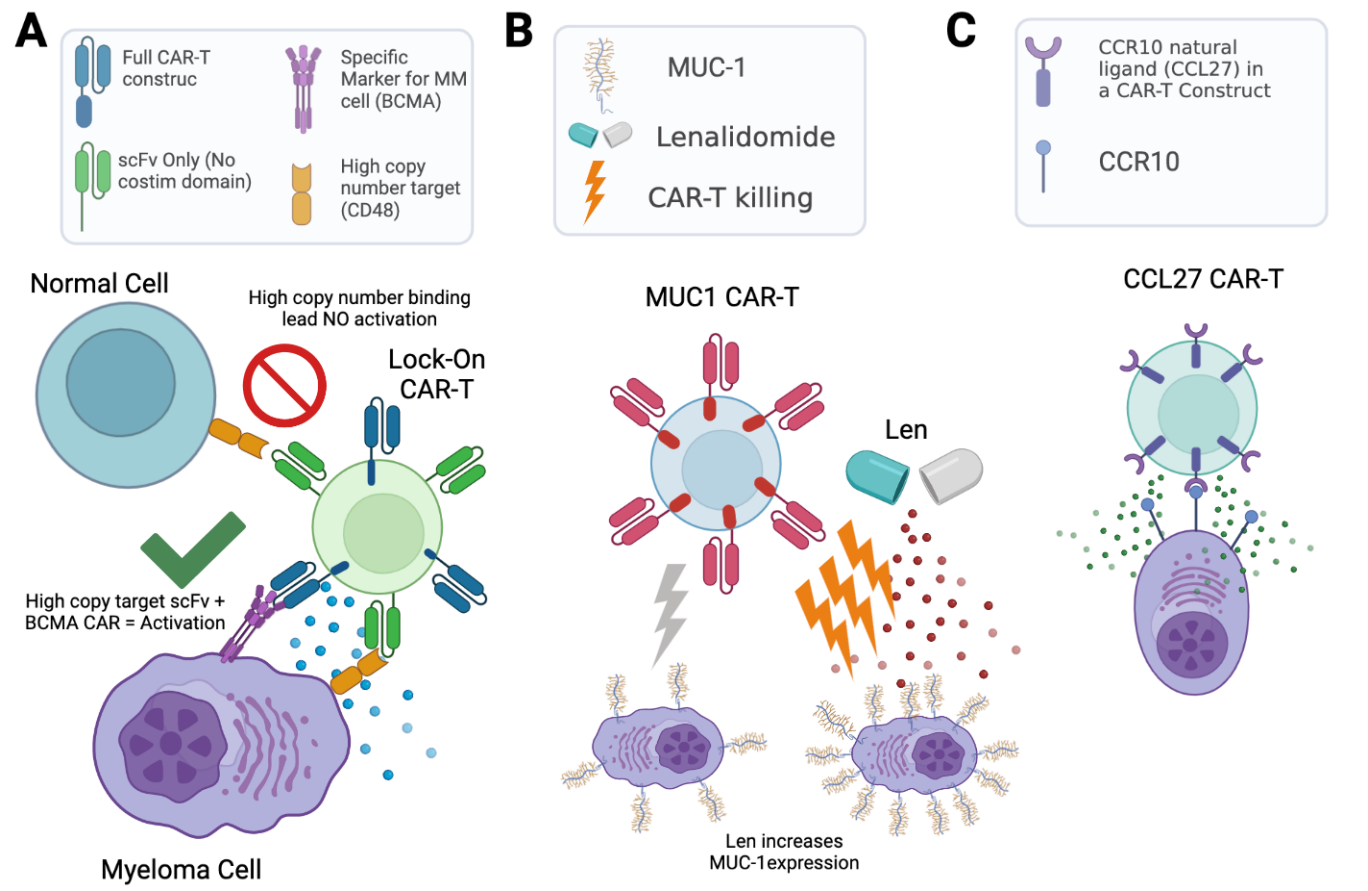
FIGURE 2: Three different engineered cell therapy strategies based on proteomic analysis. A. A “Locking-on” CAR-T concept harnesses the high abundance and hematopoietic specificity of CD48 or CD38 binding to increase avidity of anti-BCMA CAR-T. B. Acute treatment with lenalidomide increases MUC-1 expression on myeloma cells, improving anti-MUC-1 CAR-T activity. C. Proof-of-concept of stand-alone CAR-T against CCR10 using its natural ligand CCL27 as an antigen recognition domain. Figure created with BioRender

The above approaches focused on the myeloma surface proteome at baseline. However, it remains unknown how the surfaceome may be altered in the context of acute drug treatment. Such alterations may suggest rational combinations of small molecules with existing immunotherapeutic agents, a strategy which has proven promising for other myeloma immunotherapies. Taking an unbiased approach, we therefore evaluated short-term treatment (48 hr) with either lenalidomide or bortezomib, finding significant alterations in dozens of surface proteins after these front-line treatments. Most intriguingly, we found that lenalidomide treatment led to upregulation of MUC1, which we validated in primary specimens treated *ex vivo*. Indeed, based on this increase in antigen density, we found that lenalidomide co-treatment improved anti-myeloma activity of MUC-1 CAR-T cells (**Fig. 2B**). Notably, MUC-1-targeting immunotherapies are currently under clinical investigation in myeloma, suggesting a possible route to clinical translation for this combination treatment.

Moving beyond acute drug treatment, it is also an open question as to how the myeloma surfaceome is altered in the context of chronic drug resistance. Essentially all myeloma patients become refractory to both proteasome inhibitors and immunomodulatory agents. In principle, surface protein alterations may suggest immunotherapeutic targets selective for resistant disease and/or tumor biomarkers detectable by flow cytometry – an assay already used routinely in the clinical laboratory – to predict early relapse. Integrating surfaceomic data across cell lines resistant to either the proteasome inhibitors bortezomib or carfilzomib, we found common signatures of CD50, CD361/EVI2B, CD53, and ITGB7 decrease, while CD10 and CD151 were increased. In contrast, lenalidomide resistant models demonstrated consistent increase of CD33 and CD45. These protein-level changes in cell line models were also largely consistent with RNA-level alterations seen in paired diagnosis-first relapse patient tumors in the CoMMpass database. Notably, CD151 is being explored as an immunotherapy target for solid tumors, and CD33 for acute myeloid leukemia. Though more work is needed, both may therefore carry utility for targeting in myeloma. Other observed antigenic changes may serve as early markers of drug resistance.

Finally, we returned to our baseline proteomic data to discover potential stand-alone immunotherapy targets. We integrated our surface proteomic data with existing transcriptional databases describing gene expression in normal hematopoietic cells and other body tissues, relative expression levels in cancer cell lines, and computational predictions as to whether a given gene product is cell-surface localized. Developing a ranking system for the most favorable targets, with most chance of efficacy but least predicted “on target, off tumor” toxicity, we found BCMA and several other clinically-investigated targets as top-ranked antigens, an encouraging validation of our approach. However, our system also ranked highly several other previously unexplored antigens, including CCR10, TXNDC11, and LILRB4. We specifically validated that primary plasma cells express high levels of CCR10, while this gene was not detectably expressed on normal peripheral blood mononuclear cells. To generate a proof-of-principle CAR-T cell versus CCR10, we used the sequence of its natural ligand CCL27 to engage the antigen. We lentivirally transduced donor T-cells with a 2^nd^ generation CAR construct including a 4-1BB co-stimulatory domain. To overcome CCR10 induction during T-cell activation, and resulting “fractricide” in CAR-T manufacturing, we used *CCR10* knockout by CRISPR/Cas9 in donor T-cells prior to lentiviral transduction. With this approach, our CCL27-based anti-CCR10 CAR-Ts could eliminate MM.1S myeloma cells *in vitro*. Though follow-up work is needed, this finding demonstrates a promising potential new therapeutic strategy for myeloma (**Fig. 2C**).

Going forward, given that cell line models are only partially predictive of human tumor phenotypes, a major question is whether we can expand these methods to more broadly profile primary patient tumor samples. We therefore developed a “single pot”, miniaturized approach to reduce the sample input required for cell surface proteomics, characterizing >500 surface proteins in primary sample inputs of <5e6 cells. In terms of quantitative abundance, we found a good (Pearson *R* = 0.51) correlation between surface antigens found on primary samples and those in our cell line surfaceome studies. Our long-term goal is fur ther optimizing these strategies to more routinely profile the surfaceome of myeloma tumors resistant to a wide array of small molecule and immunotherapeutics.

In conclusion, cell surface proteomics is a promising and emerging method for application in cancer. Our work seeks to demonstrate a paradigm for characterizing the cancer cell surface proteome both at baseline and in the context of small molecule therapeutic treatment, illustrating how these studies can identify new therapeutic targets and potential biomarkers. We look forward to these methods being applied more widely to primary patient samples, not only in cancer but potentially in other disease states.

